# Complete Genome Sequence of the Plant Growth-Promoting Bacterium *Hartmannibacter diazotrophicus* Strain E19^T^

**DOI:** 10.1155/2019/7586430

**Published:** 2019-09-09

**Authors:** Christian Suarez, Stefan Ratering, Torsten Hain, Moritz Fritzenwanker, Alexander Goesmann, Jochen Blom, Trinad Chakraborty, Boyke Bunk, Cathrin Spröer, Jörg Overmann, Sylvia Schnell

**Affiliations:** ^1^Institute of Applied Microbiology, IFZ, Justus Liebig University Giessen, 35392 Giessen, Germany; ^2^Institute of Medical Microbiology, BFS, Justus Liebig University Giessen, 35392 Giessen, Germany; ^3^Bioinformatics and Systems Biology, Justus Liebig University Giessen, 35392 Giessen, Germany; ^4^Leibniz Institute DSMZ-German Collection of Microorganisms and Cell Cultures, 38124 Braunschweig, Germany

## Abstract

Strain E19^T^ described as *Hartmannibacter diazotrophicus* gen. nov. sp. nov. was isolated from the rhizosphere of *Plantago winteri* from a natural salt meadow in a nature protection area. Strain E19^T^ is a plant growth-promoting rhizobacterium able to colonize the rhizosphere of barley and to promote its growth only under salt stress conditions. To gain insights into the genetic bases of plant growth promotion and its lifestyle at the rhizosphere under salty conditions, we determined the complete genome sequence using two complementary sequencing platforms (Ilumina MiSeq and PacBio *RSII*). The E19^T^ genome comprises one circular chromosome and one plasmid containing several genes involved in salt adaptation and genes related to plant growth-promoting traits under salt stress. Based on previous experiments, ACC deaminase activity was identified as a main mechanism of E19^T^ to promote plant growth under salt stress. Interestingly, no genes classically reported to encode for ACC deaminase activity are present. In general, the E19^T^ genome provides information to confirm, discover, and better understand many of its previously evaluated traits involved in plant growth promotion under salt stress. Furthermore, the complete E19^T^ genome sequence helps to define its previously reported unclear 16S rRNA gene-based phylogenetic affiliation. *Hartmannibacter* forms a distinct subcluster with genera *Methylobrevis*, *Pleomorphomonas*, *Oharaeibacter*, and *Mongoliimonas* subclustered with genera belonging to *Rhizobiales*.

## 1. Introduction

Salinization of agricultural soils is a major concern causing global annual costs by loss in crop production in the order of 27.3 billion US$ [1]. Salinity causes nutritional imbalance in plant growth, development, and yield [2]. The term plant growth-promoting rhizobacteria (PGPR) is used to define bacteria that colonize the rhizosphere and stimulate plant growth [3]. Their use is a promising agricultural practice to help crops to tolerate higher salt concentrations [4]. Enhancement of plant nutrient uptake, production of 1-aminocyclo -propane-1-carboxylate (ACC) deaminase, production of phytohormones, induction of systemic tolerance, ion homeostasis mediation, induction of antioxidative enzymes, increase of osmolyte accumulation, and production of bacterial extracellular polymeric substances are among PGPR mechanisms detected to influence plant growth under salt stress [4]. The analyses of complete genomes and the gene content contribute to the understanding of physiology, ecology, and evolution of organisms [5, 6]. Genome comparison of PGPR allows insights into the mechanisms of root colonization and plant growth promotion [6–9].

Strain E19^T^ was isolated from the rhizospheric soil of *Plantago winteri* Wirtg. as part of an extensive search for PGPR from the rhizosphere of salt-resistant plant species inhabitant on a salt meadow located in a nature protection area near Münzenberg, Hesse, Germany [9]. Based on a polyphasic taxonomic approach, strain E19^T^ was previously described as a novel bacterial genus and species [10]. Among its PGP abilities, tested in pure culture, strain E19^T^ can solubilize insoluble phosphate, fix nitrogen, and lower the plant ethylene level. Strain E19^T^ root colonization capability, ACC deaminase activity *in vivo*, and plant growth promotion in summer barley plants have been confirmed under salt stress conditions [11]. Sequencing the genome of strain E19^T^ helped us to verify almost all previously reported physiological features and to compare its attributes with other PGPR under salt stress providing an insight to the molecular determinants required for PGPR under salt stress. Draft genomes with low completeness are not reliable for phylogenomics, genome synteny, genome structural, and pangenomic studies because they could contain incomplete and/or incorrect genomic data producing biased results [12]. The complete genome sequence, annotation, and data analysis of *H. diazotrophicus* strain E19^T^ contribute to clarify its unusual taxonomical classification and its suitability for large-scale genome studies including unclassified *Alphaproteobacteria.*

## 2. Material and Methods

### 2.1. Bacterial Growth and DNA Extraction

*H. diazotrophicus* E19^T^ was grown aerobically on half marine concentration agar (1.5% NaCl *w*/*v*) [10] for 72 h at 28°C; then a single colony was inoculated to 50 ml of half marine broth and grown on a gyratory shaker for 48 h at 28°C. Subsequently, 5 ml was used to inoculate 500 ml of half marine broth in a gyratory shaker for 48 h at 28°C. Cell biomass was centrifuged at 4,225 *g* at 4°C for 15 min, resuspended in MgSO_4_ 30 mM, and centrifuged at 4,225 *g* at 4°C for 15 min. 0.5 g of biomass were used for genomic DNA extraction.

### 2.2. Genome Sequencing and Annotation

Genomic DNA of *H. diazotrophicus* PGPR E19^T^ was isolated using QIAGEN Genomic Tip/100 G Kit (Hilden, Germany) and PureLink Genomic DNA Mini Kit (Life Technologies, USA). For PacBio complete genome sequencing, the SMRTbell™ template library was prepared according to the instructions from Pacific Biosciences (Menlo Park, CA, USA) following the Procedure & Checklist-20 kb Template Preparation using the BluePippin™ Size-Selection System. Briefly, for preparation of 15 kb libraries, ~8 *μ*g genomic DNA libraries was sheared using g-tubes™ from Covaris (Woburn, MA, USA) according to the manufacturer's instructions. DNA was end repaired and ligated overnight to hairpin adapters applying components from the DNA/Polymerase Binding Kit P6 from Pacific Biosciences. Reactions were carried out according to the manufacturer's instructions. BluePippin™ Size-Selection was performed according to the manufacturer's instructions (Sage Science, Beverly, MA, USA). Conditions for annealing of sequencing primers and binding of polymerase to the purified SMRTbell™ template were assessed with the calculator in RS Remote (Pacific Biosciences). SMRT sequencing was carried out on PacBio *RSII* (Pacific Biosciences) taking one 240-minute movie. Sequencing yielded 90,354 reads with roughly one gigabase of sequence information. After filtering and mapping, 82,027 reads remained with ~770 megabases, resulting in a 137x coverage of the strain E19^T^ genome. Genome assembly was performed with the RS_HGAP_Assembly.3 protocol included in SMRT Portal version 2.3.0. Two contigs were assembled from which the chromosome was trimmed, circularized, and adjusted to *dnaA* as the first gene.

Additionally, a Nextera XT paired-end library was prepared and sequenced on a MiSeq system using v2 chemistry, according to protocols provided by the manufacturer (Illumina, Netherlands). 2,455,240 paired-end reads with an average length of 210.78 bases were obtained. Quality improvement of the PacBio HGAP assembly was performed using the Burrows-Wheeler Aligner (BWA) using bwa aln and bwa sampe [13] mapping the Illumina reads onto the obtained chromosome and plasmid sequences with subsequent variant and consensus calling using VarScan2 [14] and GATK [15]. A final quality score of QV60 was attained. Automated genome annotation was carried out using Prokka [16]. For all CDSs annotated by Prokka, additional BLAST comparisons against the Swiss-Prot database [17] and against the TrEMBL database [18] were computed and the results were stored in the annotation software GenDB 2.4 [19]. The observations gained in the BLAST comparisons were used for automatic and manual refinement and curation of the Prokka annotations using the GenDB 2.4 annotation software. Comparative genome analysis was done using a generic orthology cutoff based on BLAST score ratio values [20] as implemented in EDGAR 2.3 [21].

### 2.3. Phylogenomics Analysis

To elucidate of the position of *H. diazotrophicus* E19^T^ in the tree of life, we used the PhyloPhlAn software [22] to integrate our genome into a precomputed phylogenetic tree of 3,171 microbial organisms. For a more detailed phylogeny, a core genome-based phylogenetic tree was constructed using the EDGAR platform [23]. Initially, the core genome of the 41 genomes included in this study was calculated. Subsequently, the amino acid sequences of all 288 individual gene sets of the core genome were aligned using the MUSCLE software [24]. The multiple alignments were then concatenated, resulting in a multiple alignment of 11,808 protein sequences, comprising 98,065 amino acid residues per genome, 4,020,665 in total for all 41 genomes. A phylogenetic tree was derived from this alignment using the approximate maximum likelihood approach implemented in the FastTree tool [25]. FastTree provides local support values for every branch of the tree based on the Shimodaira-Hasegawa test [26].

In order to compare the method used to calculate the core genome-based tree, a second tree was calculated using the neighbor-joining method based on a Kimura distance with bootstrap support values (200 iterations) as implemented in PHYLIP. The average nucleotide identity (ANI) was calculated using EDGAR, and OrthoANI (average nucleotide identity by orthology) was calculated using the Orthologous Average Nucleotide Identity Tool [27]. The comparative view of the gene neighborhood, showing the location and context of a set of orthologous CDSs, was analyzed using EDGAR [23].

### 2.4. Phosphate Solubilization Assay

*H. diazotrophicus* E19^T^ was grown aerobically for 7 days in SRSM1 broth [10] added with NaCl 1% and supplemented with 0.5% of rock phosphate and calcium triphosphate, respectively. Soluble phosphate was measured according to Murphy and Riley [28].

## 3. Results

### 3.1. Genomic Features

The genome of *Hartmannibacter diazotrophicus* strain E19^T^ contains one circular chromosome (5,327,443 bp) that comprises 4,868 coding sequences (CDSs) and one circular plasmid-designated HDIAp1 (122,332 bp) with 113 CDSs (Table 1). Classification into the 21 families of COG (clusters of orthologous groups of proteins) resulted in 3,999 (82.1%) and 89 (78.8%) of CDSs from the E19^T^ chromosome and plasmid, respectively (Table 2). Two copies of LSU and SSU ribosomal RNA (rRNA) genes and 51 tRNA genes representing 20 amino acids were identified in the *H. diazotrophicus* E19^T^ genome (Table 1 and [Supplementary-material supplementary-material-1] Table).

### 3.2. Classification and Phylogenetic Analysis

Based on the average nucleotide identity (ANI) and OrthoANI (average nucleotide identity by orthology), the genome of *H. diazotrophicus* E19^T^ is most similar to those of *Methylobrevis pamukkalensis* PK2 (76.27%/77.80%), *Oharaeibacter diazotrophicus* DSM 102969 (72.52%/74.44%), *Pleomorphomonas* sp. SM30 (72.48%/74.60%), *Mongoliimonas terrestris* MIMtkB18 (72.04%/73.73%), *Pleomorphomonas koreensis* DSM23070 (71.49%/73.67%), and *Kaistia adipata* DSM 17808 (70.24%/72.29%) ([Supplementary-material supplementary-material-1] Table).

**Figure 1:**
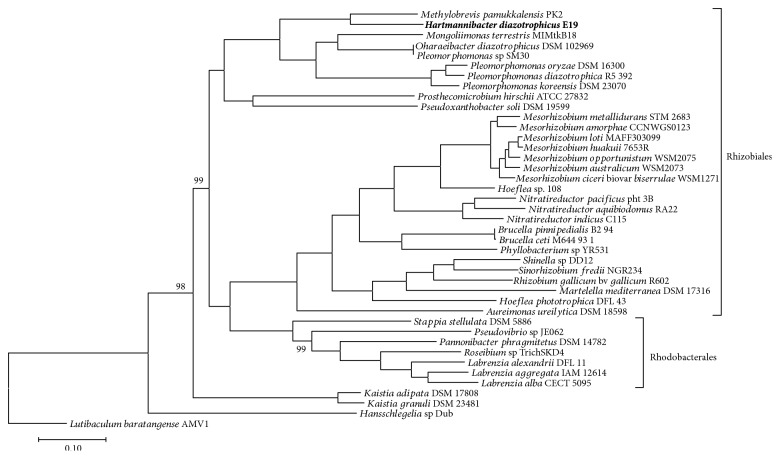
Phylogenetic tree (maximum likelihood algorithms) based on core gene sequences of strain E19^T^ and the available closest gene sequence of members of the related families of the orders *Rhizobiales* and *Rhodobacterales* and representatives of the class *Alphaproteobacteria*. Support values are shown in tree branches, when not shown values correspond to 100.

The genome assembly integrated into the precomputed phylogenetic tree using PhyloPhlAn placed *H. diazotrophicus* E19^T^ between two clusters comprised by members of the *Rhizobiales* families ([Supplementary-material supplementary-material-1] Fig). Core gene sequence-based phylogenetic trees calculated with the maximum likelihood (Figure 1) and the neighbor-joining ([Supplementary-material supplementary-material-1] Fig) algorithms with the available closest genome sequences showed that strain E19^T^ forms a distinct subclade inside members belonging to order *Rhizobiales* together with strains belonging to genera *Methylobrevis*, *Pleomorphomonas*, *Mongoliimonas*, and *Oharaeibacter.* The maximum likelihood tree resulted in nearly all support values in the core gene sequence-based phylogenetic tree being at the perfect value of 100%, with only three branches showing slightly lower values of 99.8% and 98.7%. The neighbor-joining method bootstrap support values (200 iterations) showed 100% branch conservation values in most branches. 5 branches showed bootstrap support values between 48.6 and 97.6%.

### 3.3. Genes of Central Metabolism and Cellular Processes

**Figure 2:**
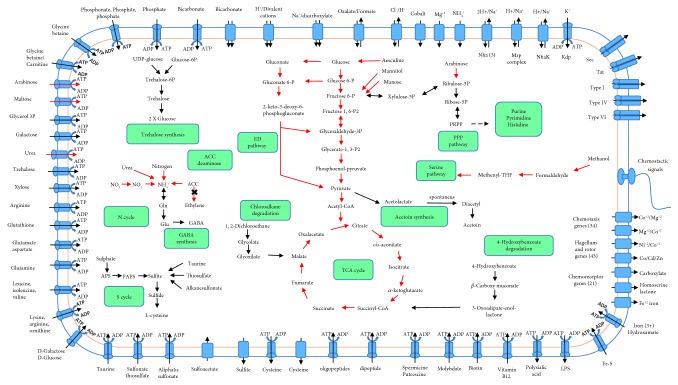
Schematic overview of metabolic pathways and transport systems found as complete gene components in the genome annotation of *H. diazotrophicus* E19^T^. With red arrows, metabolic reactions are marked that have been tested in bacterial growth or cell culture experiments [11, 12].

Carbohydrate degradation pathways Emden-Meyerhof pathway and Entner-Doudoroff pathway for glucose, arabinose, mannose, mannitol, esculin, and the respective transport systems have been detected. All genes of the TCA cycle were present (Figure 2, [Supplementary-material supplementary-material-1] Table). Exo- and polysaccharide biosynthesis and the respective transporter have been discovered ([Supplementary-material supplementary-material-1]). Flagellum and rotor genes including chemotaxis genes and chemoreceptor genes were found (Figure 2, [Supplementary-material supplementary-material-1] Table). A comprehensive list of detected genes is given in the supplementary material.

### 3.4. Genes Associated with Plant Growth Promotion Traits

Previous phenotypical and PGP abilities, tested in pure culture and in plant experiments under salt stress, were supported by the E19^T^ genome content (Figure 2, [Supplementary-material supplementary-material-1] Table) [10–12]. The E19^T^-detailed genomic content of their confirmed PGP abilities and other possible mechanisms involved in plant promotion were analyzed and described here.

### 3.5. Nitrogen Fixation

It was demonstrated previously that *H. diazotrophicus* E19^T^ is able to grow in nitrogen-free media and to reduce acetylene to ethylene, an indirect method to determine nitrogenase activity [10] ([Supplementary-material supplementary-material-1] Table). Strain E19^T^ contains genes *nifH*, *nifD*, and *nifK* encoding for the nitrogenase structural subunits, genes *nifE*, *nifN*, *nifX*, *nifB*, *nifQ*, and *nifV* involved in the synthesis and insertion of FeMo-Co into nitrogenase, *nifU*, *nifS*, and *nifZ* associated in the synthesis of metalloclusters, *nifW* described in nitrogenase protection and stabilization, *nifT* with unknown function, and *nifA*, *fixL*, *fixJ*, *fixK*, *fixN*, and *rpoN* take part in gene regulation [14, 15]. The *fixABCX* gene cluster encoding for a putative membrane complex participating in electron transfer to nitrogenase was also detected [29] ([Supplementary-material supplementary-material-1] Table).

### 3.6. Phosphate Solubilization

The ability of *H. diazotrophicus* E19^T^ to grow in mineral media with different insoluble sources of phosphate was demonstrated previously [10]. Also, as part of this study, the ability of E19^T^ to solubilize rock phosphate and tricalcium phosphate in liquid culture by measuring soluble phosphate in the supernatant was determined (0.15 and 0.37 mg·L^−1^, respectively) (Table 3). Gluconic acid (GA) is one of the major organic acids produced by bacteria able to solubilize mineral phosphates [30], and its product is catalyzed by the enzyme glucose dehydrogenase (GDH) and its cofactor pyrroloquinoline quinine (PQQ) [18, 20]. The genome of E19^T^ contained the PQQ biosynthetic *pqqBCDE* genes, but no gene encoding for GDH. Absence of the *gdh* gene could be substituted by *yliI* encoding a PQQ cofactor-dependent soluble aldose sugar dehydrogenase, which is able to oxidase glucose to gluconolactone with subsequent hydrolysis to gluconic acid [31].

Microbial enzymes catalyzing organic phosphate solubilization can be grouped into three groups: nonspecific acid phosphatases, phytases, and C-P lyases [32]. Strain E19^T^ carries genes encoding for acid phosphatase (PAP2), phosphatidic acid (PA) phosphatase-like phosphoesterase, alkaline phosphatase (phytase like), the *phn* operon encoding the C-P lyase system [33], exopolyphosphatase, and aryldialkylphosphatase. Furthermore, the presence of genes for phosphonoacetate hydrolase (PhnA) [34] and phosphonoacetaldehyde hydrolase (PhnX) [35] enables E19^T^ to convert phosphonates to phosphate in soil [36] ([Supplementary-material supplementary-material-1] Table). Phosphate uptake in strain E19^T^ occurs by the presence of the phosphate starvation system (Pst) and the phosphonate transporter system (Phn) encoded by *pstSCAB* and *phnCDE*, respectively ([Supplementary-material supplementary-material-1] Table). Additionally, *phnS*, *phnV*, *phnU*, and *phnS* encoding for the 2-aminoethylphosphonate (AEP) transporter were also found.

### 3.7. Sulfur Metabolism

The sulfur metabolism in *H. diazotrophicus* E19^T^ consists of assimilation of inorganic sulfate and mineralization of organic sulfonates. The E19^T^ genome carries 80 CDSs encoding for thiosulfate, sulfate, alkanesulfonate, and taurine transporters and genes involved in sulfur metabolism ([Supplementary-material supplementary-material-1] Table). Several copies of ABC transporters, transferring both sulfate and thiosulfate, are located on the chromosome. Plasmid HDIAp1 contains a complete sulfate ABC transport system (*cysP*, *cysT*, *cysW*, and *cysA*). Intracellularly, E19^T^ activates sulfate into adenylyl sulfate (APS) with the product of gene *cysD* and then into 3′-phosphoadenylyl sulfate (PAPS) with a bifunctional enzyme encoded by *cysNC*. Afterwards, *cysQ* mediates the conversion of PAPS into APS. Further reduction of PAPS into sulfite and hydrogen sulfide is catalyzed by *cysH* and *sir*. The E19^T^ chromosome contains eight CDSs for sulfite exporter TauE/SafE.

Alkanesulfonates are considered to be key components of organosulfur compounds in agricultural soils [37]. Alkanesulfonates are transported by aliphatic sulfonate ABC transport (*ssuABC*) into the cell and then converted into sulfite with the enzymes alkanesulfonate monooxygenase (*ssuD*) and a NADPH-dependent FMN reductase (*ssuE*) [38]. The E19^T^ chromosome contains several copies of the sulfonate *ssuABC* transporter, but neither *ssuD* genes nor *ssuE* genes. Interestingly, plasmid HDIAp1 contains another set of CDSs for *ssuABC* and *ssuE*. Additionally, the E19^T^ genome encodes the *tauABC* genes responsible for taurine (aliphatic organosulphonate) transport inside the cell for further degradation into alanine and sulfoacetaldehyde by the enzyme taurine-pyruvate aminotransferase (Tpa) [39]. Moreover, *cysB* genes involved in sulfur regulation in gram-negative bacteria and the genes cysteine synthase (*cysK*) and O-acetylserine sulfhydrylase (*cysK*1) for cysteine biosynthesis from sulfide were identified in the genome of E19^T^ [7, 29] ([Supplementary-material supplementary-material-1] Table).

### 3.8. ACC Deaminase

One of the mechanisms of PGPR to alleviate salt stress is the synthesis of the enzyme 1-aminocyclo-propane-1-carboxylate (ACC) deaminase or its homologue D-cysteine desulfhydrase encoded by *acdS* or *dcyD*, respectively. Both enzymes lower ethylene accumulation in stressed plants by cleaving ACC, an immediate precursor of ethylene in plants, to form ammonia and *α*-ketobutyrate [30, 40]. This reaction is pyridoxal phosphate dependent, and both ACC deaminase and D-cysteine desulfhydrase belong to the pyridoxal phosphate-dependent enzyme family PALP. In the *H. diazotrophicus* E19^T^ genome, neither *acdS* genes nor *dcyD* genes are present but eight CDSs containing genes encoding genes belonging to the PALP domain ([Supplementary-material supplementary-material-1] Table) have been found. Among these genes, catabolic L-threonine dehydratase (TdcB) and diaminopropionate ammonia-lyase (YgeX) both show lyase activity and potentially perform ammonia synthesis similarly to the enzymes encoded by *acdS* and *dcyD*.

### 3.9. Methylotrophy

The ability of *H. diazotrophicus* E19^T^ to grow using methanol as a carbon source was tested in the presence and absence of lanthanum as similarly described [41]. No growth with methanol and without La was observed (unpublished results). The E19^T^ genome contains genes involved in alpha-proteobacterial methylotrophy such as PQQ-dependent methanol dehydrogenase (MDH) and PQQ synthesis and genes of metabolic pathways such as the H4F pathway, H4MPT pathway, formate oxidation, and serine cycle ([Supplementary-material supplementary-material-1] Table). Among the previously described PQQ-dependent methanol dehydrogenase (MDH) genes *xoxF* and *mxaF* in closely related genomes [42], *H. diazotrophicus* E19^T^ contains three CDSs encoding for methanol dehydrogenase containing genes related to methanol oxidation ([Supplementary-material supplementary-material-1] Table). CDSs 2042 and 2631 showed BLASTP percent identity to a PQQ-dependent methanol dehydrogenase of *Methylobrevis pamukkalensis* PK2 corresponding at 95.2% and 81.2%, respectively. CDS 3031 showed 86.2% percent identity to iron-containing alcohol dehydrogenase of *Methylobrevis pamukkalensis* PK2. The gene synteny for CDS 2042, *moxF*, showed a conserved genomic neighborhood among closely related genomes. On the contrary, CDSs 2631 and 3031 showed a nonconserved genomic neighborhood ([Supplementary-material supplementary-material-1] Fig).

### 3.10. Volatile Organic Compounds

Acetoin and 2,3-butanediol, two growth-promoting VOCs, are synthesized by the condensation of two pyruvate molecules into acetolactate. Acetolactate is produced by the enzyme acetolactate synthase, then acetolactate is decarboxylated to acetoin by the enzyme acetolactate decarboxylase, and finally, 2,3-butanediol is obtained from the reduction of acetoin by the enzyme acetoin reductase. The E19^T^ genome contains *ilv* genes encoding for acetolactate synthase but neither genes for acetolactate decarboxylase nor genes for acetoin reductase are present. E19^T^ could be able to synthetize acetoin by a spontaneous decarboxylation of acetolactate, in the presence of oxygen, into diacetyl and its subsequent reduction to acetoin by the enzyme diacetyl reductase encoded by *budC* [43] ([Supplementary-material supplementary-material-1] Table).

### 3.11. Iron Acquisition

Siderophore translocation through the bacterial outer membrane is performed by an energy-transducing complex with proteins TonB, ExbB, and ExbD [44]. Once in the periplasmic space, the ferric siderophore binds to its cognate periplasmic-binding protein and is then actively transported across the cytoplasmic membrane by an ATP transporter system. In *H. diazotrophicus* E19^T^, the genes *tonB*, *exbB*, and *exbD* were identified. Moreover, the iron(III)-hydroxamate ABC transporter cluster *fhuCDB* and the ferric hydroxamate uptake gene *fhuA*, responsible of transport of ferrichrome and other Fe^3+^-hydroxamate compounds, are present (Fe^3+^-aerobactin, Fe^3+^-coprogen) [45]. Furthermore, the E19^T^ genome contains CDS for the chelated iron transport system cluster *yfeABCD* and ferrous iron uptake protein *efeU* ([Supplementary-material supplementary-material-1] Table).

### 3.12. Genes Involved in Salt Tolerance

Rhizobacteria accumulate compatible solutes such as trehalose, glutamate, proline, and glycine betaine during osmotic stress [33, 34]. The strain E19^T^ genome contains *otsA* and *otsB* encoding for trehalose-6-phosphate synthase and trehalose-phosphatase, both needed to catalyze GDP- or UDP-glucose conversion to trehalose [46]. Synthesis of glycine betaine acting as compatible solute under salt stress conditions is known in plants and halophilic bacterial strains [36, 37]. The strain E19^T^ genome contains *betA* and *betB* genes, encoding for choline dehydrogenase and betaine aldehyde dehydrogenase, key genes for glycine betaine synthesis [38, 39]. Also, genes encoding biosynthesis of proline and glutamate are present in the E19^T^ genome ([Supplementary-material supplementary-material-1] Table).

The E19^T^ genome carries heat shock genes *dnaJ*, *dnaK*, *groES*, *groEL*, *htpG*, *htpX*, *hspQ*, *grpE*, and *ibpA* and the cold shock gene *cspA* ([Supplementary-material supplementary-material-1] Table). Moreover, the *clpB* gene, a heat shock protein, specified to be upregulated during salt stress in marine bacteria [47] is also contained. The E19^T^ genome carries CDSs encoding for peroxidases, superoxidase, and glutathione S-transferase ([Supplementary-material supplementary-material-1] Table). These genes play a role in the protection of cell oxidative stress caused by salt stress [48].

PGPR-inoculated plants are able to increase K^+^ concentration, which in turn resulted in a high K^+^ Na^+^ ratio, influencing plant salinity tolerance [4]. The *H. diazotrophicus* E19^T^ genome contains the *kdp* operon which encodes the high-affinity K^+^ uptake [49]; two CDSs encoding protein Kup and genes *trkG* and *trkA* are part of constitutive potassium transport systems [50] ([Supplementary-material supplementary-material-1] Table).

The strain E19^T^ genome carries CDSs encoding for protein Na^+^/H^+^ antiporters (Nha) responsible for importing H^+^ and pumping out Na^+^ and the gene cluster *mrpABCDEFG* encoding a Na^+^/H^+^ antiporter that plays a role in Na^+^ extrusion, pH homeostasis, cell volume regulation, and establishment of an electrical potential of Na^+^ in bacteria [41–43].

### 3.13. Secretion Systems

The strain E19^T^ genome contains CDSs for genes encoding for potential protein secretion systems types I, II, III, IV, and VI; Tat; and Sec ([Supplementary-material supplementary-material-1] Table) (Figure 2). The majority of the CDSs of secretory protein systems correspond to systems Sec, Tat, and types I and VI the in E19^T^ genome. CDSs encoding genes *tolC*, *hlyD*, *prsD*, *psrE*, and *exsH* and CDSs *vgrGA*, *hcp*1, *icmF*, and *lmpA* encoding for components of secretion system types I [51] and VI [52], respectively, were found in the E19^T^ chromosome. *trb* genes encoding for a type IV secretion system [50, 53] were found on both the E19^T^ chromosome and plasmid HDIAp1.

### 3.14. Resistance to Heavy Metals and Degradation of Aromatic Compounds

There are protein-coding genes in the E19^T^ genome like *copD*, *rcnA*, *czcD*, *arsC*, and *merA* involved in resistance mechanisms to copper, cobalt, nickel zinc, arsenate, mercury, and cadmium ([Supplementary-material supplementary-material-1] Table).

63 CDSs are found for catabolism of protocatechuate (*pca* genes), catechol (*cat* genes), naphthalene (*nag* genes), and toluene and degradation of ring cleavage products to TCA cycle intermediates derived from 4-hydroxybenzoate ([Supplementary-material supplementary-material-1] Table). Moreover, E19^T^ contains genes encoding for nitrilase, nitroreductase, fluoroacetate dehalogenase, and haloacid dehalogenases recognized as contaminant-degrading enzymes in rhizoremediation processes [54].

### 3.15. Colonization Traits and Other Genes Involved in Plant Growth Promotion

All genes known to be required for the synthesis of functional flagella [55] are present, confirming previously observed strain E19^T^ motility [10]. Also, the E19^T^ genome contains genes involved in chemotaxis and *quorum sensing*. Furthermore, plasmid HDIAp1 carries genes encoding for wall-associated protein A (*wapA*), a protein found to inhibit the growth of neighboring cells that could benefit on the E19^T^ root colonization ability [56] ([Supplementary-material supplementary-material-1] Table). The strain E19^T^ genome contains *phzF* and *phzD* encoding phenazine biosynthesis [57] and *gabD* and *gabT* encoding for synthesis of *γ*-aminobutyric acid (GABA) ([Supplementary-material supplementary-material-1] Table).

### 3.16. Plasmid-Associated Genes

Plasmid HDIAp1 contains CDSs for *repABC* genes, in accordance with many plasmids in *Alphaproteobacteria* [58]. F-type *tra*/*trb* T4SS genes responsible for plasmid conjugal transfer [50, 53] are found in plasmid HDIAp1 and the E19^T^ chromosome ([Supplementary-material supplementary-material-1] Table).

## 4. Discussion

The complete genome sequence and analysis of *H. diazotrophicus* E19^T^ isolated from the rhizosphere of *Plantago winteri* Wirtg. are presented in this study and were compared to the phenotypic characteristics and plant growth-promoting abilities of previous studies (Figure 2, [Supplementary-material supplementary-material-1] and [Supplementary-material supplementary-material-1] Tables). An accurate orthology inference is essential at related species comparative genomic researches. Majority of the available tools for orthology inference have difficulties in accuracy at the automatization of the process without requiring manual intervention [54, 59]. A special manual gene curation effort was made to annotate genes related to plant growth promotion traits, due to the annotation detail incompleteness of their closely related bacterial genomes.

Our previous taxonomic description [10] and results of greenhouse experiments with barley plants under salt stress [10, 12] are supported by the genome data of strain E19^T^ (Table 3, [Supplementary-material supplementary-material-1] and [Supplementary-material supplementary-material-1] Table). Barley plants (*Hordeum vulgare* L.) inoculated with strain E19^T^ in nonsterile soil under salt stress conditions significantly increased root and shoot dry weights, water content in the root system, and the root-to-shoot ratio [11]. Moreover, E19^T^ inoculation decreased root sodium uptake compared to uninoculated plants, indicating an additive effect to the barley root Na^+^ exclusion mechanism [12, 48]. Furthermore, inoculation of strain E19^T^ has shown beneficial effects on germination of alfalfa cultivars under salt stress [60].

Strain E19^T^ was isolated from a salty environment and its ability to grow under different salt concentrations was proven [10]. Accordingly, the presence of genes encoding for synthesis of glycine betaine and trehalose, the high-affinity K^+^ uptake system (Kdp), Na^+^/H^+^ antiporters, and heat and cold shock proteins are associated with the survival of microorganisms under salt or osmotic stress [28, 39, 61, 62]. Additionally, the genome of strain E19^T^ encodes various antioxidants enzymes and genes for acetoin production, mechanisms involved in bacterial-mediated plant antioxidative protection and induction of systemic tolerance during salt stress conditions [4].

Bacteria producing ACC deaminase are able to promote root elongation and plant growth by lowering ethylene levels in the roots of developing plants [63]. Previously, we have reported *H. diazotrophicus* E19^T^ ACC deaminase activity *in vivo* and its effect on the reduction of ethylene emission in inoculated summer barley seedlings exposed to salt stress conditions [11]. Neither *acdS* genes nor *dcyD* genes, known to code for enzymes with ACC deaminase activity [64], are contained in the E19^T^ genome. However, genes *tdcB* and *ygeX* can be considered as candidate genes for ACC deaminase activity since they belong to the pyridoxal phosphate-dependent enzyme family protein (PALP) and have ammonia lyase activity similar to genes *acdS* and *dcyD* ([Supplementary-material supplementary-material-1] Table). The enzymatic reaction of enzymes encoded by genes *tdcB* and *ygeX* could explain the ability of E19^T^ to grow in DF medium amended with ACC as the only nitrogen source and its ACC deaminase activity *in vitro*.

In line with traits of strain E19^T^ PGP determined in previous studies, genes were found for phosphate solubilization and nitrogen fixation ([Supplementary-material supplementary-material-1] and [Supplementary-material supplementary-material-1] Tables), well-known PGP traits and most common traits found in a comparative genome analysis of PGPR strains belonging to different genera of *Proteobacteria* [65]. The E19^T^ genome carries genes for production of gluconic acid, several enzymes involved in phosphate solubilization, and two high-affinity phosphate transporter systems, supporting the phosphate solubilization of different organic and inorganic phosphorus sources tested previously for the strain. Our previously reported nitrogen fixing capacity of strain E19^T^ [10] is supported by the presence of 14 *nif* genes and other nitrogen fixation-related genes contained in the genome.

The E19^T^ genome contains genes involved in alpha-proteobacterial methylotrophy. Interestingly E19^T^ can use methanol only in presence of La as shown previously [41]. This enzyme dependence on rare earth elements was shown recently, and the discovery in E19^T^ will help to understand the ecology of the pathway.

PGPR can positively influence plant growth through increasing the availability of sulfur and iron acquisition. Plants assimilate sulfur from soils, and its mobilization is mediated by the rhizospheric microbial community [8]. The E19^T^ genome carries genes involved in hydrogen sulfide synthesis known to influence plant growth and seed germination [19] and several genes involved in mineralization of carbon-bound sulfur that contribute with the cycling of soil organic sulfate and plant growth promotion in soils with low sulfur availability [21].

In accordance with our previous results [10], no genes coding neither for synthesis of catecholate siderophores nor for synthesis of other siderophores are contained in the E19^T^ genome. However, E19^T^ contains genes to heterologously adopt siderophores produced by other soil bacteria via various high-affinity-specific uptake ferric siderophore complexes [45] ([Supplementary-material supplementary-material-1] Table).

The genomic content of E19^T^ shows CDSs belonging to secretory protein systems Sec, Tat, and types I-IV (Figure 2) ([Supplementary-material supplementary-material-1] Table). The presence of Sec and type I, II, III, IV, and VI secretion systems in rhizobacteria has been related to bacterial-plant growth promotion by providing rhizosphere colonization ability [8]. Secretory system type I has been involved in biofilm formation and surface symbiosis recognition in PGPB *Mesorhizobium loti* and *Bradyrhizobium japonicum* [51]. Secretory system type VI has been reported to be present among several genomes of strains involved in plant-bacterial interaction including bacterial plant pathogens, bacterial-plant symbionts, and plant growth-promoting rhizobacteria [52]. Although the E19^T^ genome carries CDSs encoding for proteins associated to secretion systems of types II and III, the absence of key components of these systems suggests that they could be nonfunctional.

The presence of genes in the E19^T^ genome for motility, chemotaxis, quorum sensing, antibiotic compounds, and secretory protein systems was found ([Supplementary-material supplementary-material-1] Table). These traits have been described as bacterial traits required for rhizosphere and rhizoplane colonization [66] and support E19^T^-efficient root colonization previously evaluated in barley plantlets grown under salt stress by fluorescence *in situ* hybridization (FISH) [12, 56].

The E19^T^ genome contains many genes supporting the survival in salty soil condition, an efficient root colonization, nitrogen and phosphorus supply to the plant, and a reduction of the ethylene level in the rhizosphere. The sum of these genetic traits complements the observed growth promotion of salt-stressed plants inoculated with strain E19^T^ [12, 49]. Certainly, the annotated genome of E19^T^ will help us in the future to decipher bacterial-plant interactions under salt stress using a transcriptomics approach [67]. In particular, the expression and role of the here-proposed ACC deaminase candidate genes need to be further analyzed since the reduction of the ethylene level in barley plants under salt stress is considered as the major plant growth promoting effect of strain E19^T^.

In addition, the E19^T^ genome carries several genes encoding for contaminant-degrading enzymes and enzymes for degradation of aromatic compounds ([Supplementary-material supplementary-material-1] Table). These findings are in accordance with previously reported isolate 23-01, with 99.9% 16S rRNA gene similarity to E19^T^, able of complete mineralization of different polycyclic aromatic hydrocarbon compounds [68]. Also, coding genes involved in the resistance to heavy metals were found ([Supplementary-material supplementary-material-1] Table) that could contribute to E19^T^ environmental fitness in heavy metal-contaminated soils [7]. As previously reported, the 16S rRNA gene sequence similarity of strain E19^T^ with all available relatives showed less than 93.5% with genera members of the orders *Rhizobiales* and *Rhodobacterales* [10] and 95.2% to recently described genus *Methylobrevis* [69]. The 16S rRNA gene-based tree reconstruction located strain E19^T^ in an independent subcluster not clearly affiliated to any classified genus neither to the 13 families of the order *Rhizobiales* nor to the *Rhodobacterales* family [10].

The phylogenetic tree calculated using PhyloPhlAn placed *H. diazotrophicus* E19^T^ among *Rhizobiales* ([Supplementary-material supplementary-material-1] Fig). This result gave a good overall impression of its taxonomic position, although it is important to remark that the PhyloPhlAn phylogeny is based only on 3978 amino acid residues per genome and closely related genomes were missing in the precomputed tree of life.

The core genome-based tree reconstruction with EDGAR (Figure 1) located strain E19^T^ in an independent subcluster of the order *Rhizobiales* together with members of genera *Pleomorphomonas*, *Mongoliimonas*, *Oharaeibacter*, and *Methylobrevi*s. In contrast to PhyloPhlAn, EDGAR used 98,065 amino acid residues per genome, having 4,020,665 in total for all 41 genomes.

Strain E19^T^ shared an ANI value of 76.27% with the closest available genome sequence *Methylobrevis pamukkalensis* PK2 and less than 72.52% with *Alphaproteobacteria* available genome sequences. These ANI values are far below an ANI species boundary of 94-96% and a genus threshold of <93% [70]. Although there is not a stable ANI family threshold, due to the uneven distribution in genome sequences of strains in between families, the range of ANI family threshold values is calculated between 81.0 and 96% [71]. These results were confirmed using an improved ANI algorithm OrthoANI (average nucleotide identity by orthology) that avoids the problem of large differences in reciprocal ANI values associated with the ANI algorithm [67] ([Supplementary-material supplementary-material-1] Table). In sum, results indicated a very low taxonomy relatedness with sequenced available genomes suggesting that *H. diazotrophicus* strain E19^T^ could be in the future proposed as a new family among the order *Rhizobiales* and that it has an undescribed affiliation to strain *Methylobrevis pamukkalensis* PK2. This statement will remain unraveled until more isolates of genus *Hartmannibacter* and close-relative taxa are isolated and sequenced to perform further comparative genome studies.

The complete E19^T^ high-quality genome sequence, obtained by two complementary sequencing platforms, makes this data relevant for phylogenomics, genome structural analysis, and pangenomic approaches including unclassified *Alphaproteobacteria*. Furthermore, it contributes to plant-associated bacterial genome prediction approaches [72] in order to identify relevant traits related with plant growth promotion.

## Figures and Tables

**Table 1:** General features of the *H. diazotrophicus* E19^T^ genome.

Feature	Chromosome	Plasmid
Size (bp)	5,327,443	122,332
G+C content (%)	64.08	59.21
Number of CDSs	4868	113
Gene density (genes per kb)	1.094	1.082
CDS average length	946.5	903.3
tRNA	51	—
rRNA	6	—
Number of genes with assigned function	3917 (80.46%)	96 (84.95%)
Number of genes without assigned function	951 (19.53%)	17 (15.04%)

**Table 2:** COG functional categories of the *H. diazotrophicus* E19^T^ genome.

Type	Functional COG categories	Chromosome E19^T^	Plasmid HDIAp1
Information storage and processing	(A) RNA processing and modification	0	0
	(B) Chromatin structure and dynamins	2	0
	(J) Translation, ribosomal structure, and biogenesis	168	0
	(K) Transcription	279	8
	(L) Replication, recombination, and repair	141	18

Cellular processes and signaling	(D) Cell cycle control, cell division, and chromosome partitioning	16	0
	(M) Cell wall/membrane/envelope biogenesis	169	3
	(N) Cell motility	43	0
	(O) Posttranslational modification, protein turnover, and chaperones	161	1
	(T) Signal transduction mechanisms	211	2
	(U) Intracellular trafficking, secretion, and vesicular transport	47	7
	(V) Defense mechanisms	37	0

Metabolism	(C) Energy production and conversion	325	7
	(E) Amino acid transport and metabolism	419	8
	(F) Nucleotide transport and metabolism	73	0
	(G) Carbohydrate transport and metabolism	267	11
	(H) Coenzyme transport and metabolism	134	1
	(I) Lipid transport and metabolism	164	2
	(P) Inorganic ion transport and metabolism	219	8
	(Q) Secondary metabolite biosynthesis, transport, and catabolism	105	3
	(R) General function prediction only	306	3
	(S) Function unknown	713	7
	Total	3999	89


**Table 3:** Plant growth-promoting abilities of *H. diazotrophicus* E19^T^ as described in [11, 12] and from this study^∗^.

Plant growth-promoting abilities	Measurements
Acetylene reduction	0.18 *μ*mol N_2_ (mg protein)^−1^ h^−1^
ACC deaminase activity	
NaCl 1%	0.56 ± 0.20 *μ*mol *α*-ketobutyrate mg protein^−1^ h^−1^
NaCl 2%	1.29 ± 0.82 *μ*mol *α*-ketobutyrate mg protein^−1^ h^−1^
NaCl 3%	2.60 ± 1.2 *μ*mol *α*-ketobutyrate mg protein^−1^ h^−1^
Growth in mineral media with different P sources	Ca_3_O_8_P_2_, AlPO_4_, FePO_4_ and phytate
Soluble phosphate after 7 days of growth	
Calcium triphosphate solubilization^∗^	0.37 mg L^−1^
Rock phosphate solubilization^∗^	0.15 mg L^−1^

## Data Availability

The genome sequences of the *H. diazotrophicus* E19^T^ chromosome and plasmid HDIAp1 have been deposited to the European Nucleotide Archive under accession numbers LT960614 and LT960615, respectively.

## Supplementary Materials

Supplementary Materials S1 Figure: precomputed phylogenetic tree of life integrating the *H. diazotrophicus* E19^T^ genome calculated with PhyloPhlAn. Bootstrap values are shown in tree branches. S2 Figure: phylogenetic tree based on core gene sequences of strain E19^T^. Phylogenetic tree based on core gene sequences of strain E19^T^ and the available closest gene sequence of members of related families of the orders *Rhizobiales and Rhodobacterales* and representatives of the class Alphaproteobacteria. Bootstrap values are shown in tree branches, when not shown values correspond to 100%. S3 Figure: genomic organization of the strain E19^T^ genome coding sequences for methanol dehydrogenase homolog genes and its closest relative genomes. Same-color arrows correspond at orthologous genes among genome sequences. A) CDS 2042, B) CDS 2631, and CDS 3031. S1 Table: *H. diazotrophicus* E19^T^ average nucleotide identity (ANI) and OrthoANI (average nucleotide identity by orthology) to the next relative available genome sequences. S2 Table: *H. diazotrophicus* E19^T^ previously described phenotypical characteristics compared with its genomic content [11]. S3 Table: plant growth-promoting activities and respective genes present or absent in E19^T^. S4 Table: *H. diazotrophicus* E19^T^ nitrogen fixation-related genes. S5 Table: *H. diazotrophicus* E19^T^ phosphate-related genes. S6 Table: *H. diazotrophicus* E19^T^ sulfur-related genes. S7 Table: *H. diazotrophicus* E19^T^ CDSs containing PALP domain genes. S8 Table: *H. diazotrophicus* E19^T^ methylotrophy-related genes. S9 Table: *H. diazotrophicus* E19^T^ volatile organic compound-related genes. S10 Table: *H. diazotrophicus* E19^T^ iron acquisition-related genes. S11 Table: *H. diazotrophicus* E19T salt tolerance-related genes. S12 Table: *H. diazotrophicus* E19^T^ central metabolisms and protein secretory system-related genes. S13 Table: *H. diazotrophicus* E19^T^ heavy metal resistance-related genes. S14 Table: *H. diazotrophicus* E19^T^ aromatic compound degradation-related genes. S15 Table: *H. diazotrophicus* E19^T^ root colonization-related genes. S16 Table: *H. diazotrophicus* E19^T^ biocontrol agent and plant growth regulator-related genes. S17 Table: *H. diazotrophicus* E19^T^ tRNA and rRNA genes. S18 Table: *H. diazotrophicus* E19^T^ plasmid-related genes.
