# Supplementary material for: Complete Genome Sequence of the Plant Growth-Promoting Bacterium Hartmannibacter diazotrophicus Strain E19T
Source: Int J Genomics. 2019 Sep 9;2019:7586430. doi: 10.1155/2019/7586430 (PMC6754898; doi:10.1155/2019/7586430)

Supplementary Materials

Complete Genome Sequence of the Plant Growth-Promoting Bacterium *Hartmannibacter diazotrophicus* strain E19^{T}

Christian Suarez[1*], Stefan Ratering[1], Torsten Hain[2], Moritz Fritzenwanker[2], Alexander Goesmann[3], Jochen Blom[3], Trinad Chakraborty[2], Boyke Bunk[4], Cathrin Spröer[4] Jörg Overmann[4], and Sylvia Schnell[1]

S1 Table

H. diazotrophicus E19^{T} average nucleotide identity (ANI) and OrthoANI (Average Nucleotide Identity by Orthology) to next relative available genome sequences.

**Next relative bacteria**	**ANI (Mean)**	**OrthoANI**
Methylobrevis pamukkalensis PK2	76.27	77.80
Oharaeibacter diazotrophicus DSM 102969	72.52	74.44
Pleomorphomonas sp. SM30	72.48	74.60
Mongoliimonas terrestris MIMtkB18	72.04	73.73
Pleomorphomonas koreensis DSM 23070	71.49	73.67
Pleomorphomonas diazotrophica R5-392	70.97	72.79
Kaistia adipata DSM 17808	70.24	72.29
Kaistia granuli DSM 23481	70.00	72.00
Pleomorphomonas oryzae DSM 16300	69.97	72.28
Pannonibacter phragmitetus DSM 14782	69.77	71.49
Stappia stellulata IAM12621^{T} DSM 5886	69.73	71.58
Hoeflea sp. 108	69.5	71.69
Sinorhizobium fredii NGR234	69.46	71.79
Nitratireductor pacificus pht 3B	69.42	71.60
Brucella pinnipedialis B2 94	69.41	69.83
Mesorhizobium metallidurans STM 2683	69.37	71.55
Mesorhizobium loti MAFF303099	69.36	71.50
Mesorhizobium australicum WSM2073	69.36	71.27
Mesorhizobium opportunistum WSM2075	69.28	71.36
Mesorhizobium cicero biovar *biserrulae* WSM1271	69.24	71.20
Mesorhizobium amorphae CCNWGS0123	69.20	71.51
Mesorhizobium huakuii 7653R	69.15	71.62

Lutibaculum_baratangense AMV1	69.01	71,20
Aureimonas ureilytica DSM 18598	68.97	71.09
Martelella mediterranea DSM 17316	68.88	71.16
Labrenzia aggregata IAM 12614	68.87	70.76
Nitratireductor indicus C115	68.85	70.67
Shinella sp. DD12	68.80	71.49
Nitratireductor aquibiodomus RA22	68.79	70.56
Rhizobium gallicum bv *gallicum* R602	68.62	71.11
Brucella ceti M644 93 1	68.60	69.93
Hoeflea phototrophica DFL 43	68.44	70.11
Labrenzia alexandrii DFL 11	67.82	69.35
Phyllobacterium sp. YR531	67.43	69.53
Labrenzia alba CECT 5095	67.34	69.52

S2 Table

H. diazotrophicus E19^T previously described phenotypical characteristics compared with its genomic content [11]

**Phenotypical characteristics**	**E19^T**	**Genes known for the respective function not found in E19^T genome**	**Genes known for the respective function found in E19^T genome**
Catalase	-		*katG*
Superoxide dismutase	+		*sodB*
Casein (serine protease)	+		*deg*P
Starch	-	*mal*FG, *mal*Q, *lam*B	*amy*D, *mal*K, *mal*P
CM-cellulose	-	*cel*S, *cel*B, *cel*E	
Nitrate reduction	+		*nas*A
Urease activity	+		*ure*ABCDG
Aesculin hydrolysis	+		*bgl*X
D-glucose assimilation	+		*mgl*AB
Arabinose assimilation	+		*ara*AB, *kds*D, *ara*G, *ara*Q
Mannose assimilation	+	*man*Y, *man*Z	*mal*X, *alg*A
Mannitol assimilation	+	*mtl*A	*mtl*K
Gelatin hydrolysis (metalloproteases)	-		*fts*H, *mmp*A
Indole production	-	*tna*A	
D-glucose fermentation	-	PDC, *adh*	
Arginine dihydrolase	-		*arc*A
N-acetylglucosamine assimilation	-	*nag*E	*nag*A
Maltose	-		*mgl*AB
Gluconate	-	*gnt*P, *gnt*M *usg*A	
Caprate	-		*est*B
Malate	-	*yqk*I, *yuf*R, *mae*N	
Citrate	-	*citQRP*	
Phenylacetate	-	*paa*	
C8 esterase lipase	+		*lip*1
C14 lipase	+		*lip*1
Valine arylamidase	+	*vdh*	
Acid phosphatase	+		Pap2
Naphthol-AS-BI-phosphohydrolase	+		*suh*B
α -glucosidase	+	*agl*A	*ygj*K
N-acetyl- ß -glucosaminidase	+		*nag*Z
Alkaline phosphatase	-	*peh*A	*pho*A
C4 esterase	-	*est*A	
Leucine arylamidase	-		*pep*B
Cystine arylamidase	-	*dmp*A	
Trypsin	-	*spr*T	
α-Galactosidase	-	*gal*A	
ß -Galactosidase	-	*lac*Z	
α-Chymotrypsin	-	*ctr*B	
ß -Glucuronidase	-	*gus*A	
ß -Glucosidase	-	*ggh*A	
α-Mannosidase	-	*mng*B	
α-Fucosidase	-	*amy*E	

S3 Table

Plant growth-promoting activities and respective genes present or absent in E19^T.

PGP traits	Activity tested in E19^T	Genes	E19^T
Nitrogen fixation	Indirect test of nitrogenase activity by acetylene reduction [11]<br><br>Growth on N-free growth medium [11]	*nifHDK*	+
Phosphate solubilization	Growth on agar plates with different phosphate sources $Ca_3(PO_4)_2$, $AlPO_4$, $FePO_4$ and phytate [11]<br><br>Solubilization of rock phosphate and tricalcium phosphate in liquid culture (in this study)	*pqqBCDE*	+
		*yli*I	+
		pstSCAB	+
		*phn*CDE	+
		phnSVUS	+
		phnAX	+
		phoD	+
		phoR	+
ACC deaminase	Growth on DF medium agar supplemented with ACC [11]<br><br>ACC deaminase activity in minimal salt medium supplemented with ACC [12]<br><br>ACC deaminase activity *in vivo* by ethylene emission measurements in salt stress barley plants [12]	*acdS*	-
		dcyD	-
		*yge*X	+
		*tdc*B	+
Motility	Flagella staining [11]<br>Root colonization ability [12]	*fliGMPI flgIHG*	+
Catalase	Tested negative [11]	*katG*	-
Superoxide dismutase	Tested positive [11]	*sodB*	+
IAA production	Growth in media and activity tested with Salkowsky reagent [12]	*ipdC, ppdC*	-
Catecholate siderophore production	Tested negative [11]	*pvd*	-

S1 Figure. Precomputed phylogenetic tree of life integrating *H. diazotrophicus* E19^T genome calculated with Phylophlan. Bootstrap values are shown in tree branches

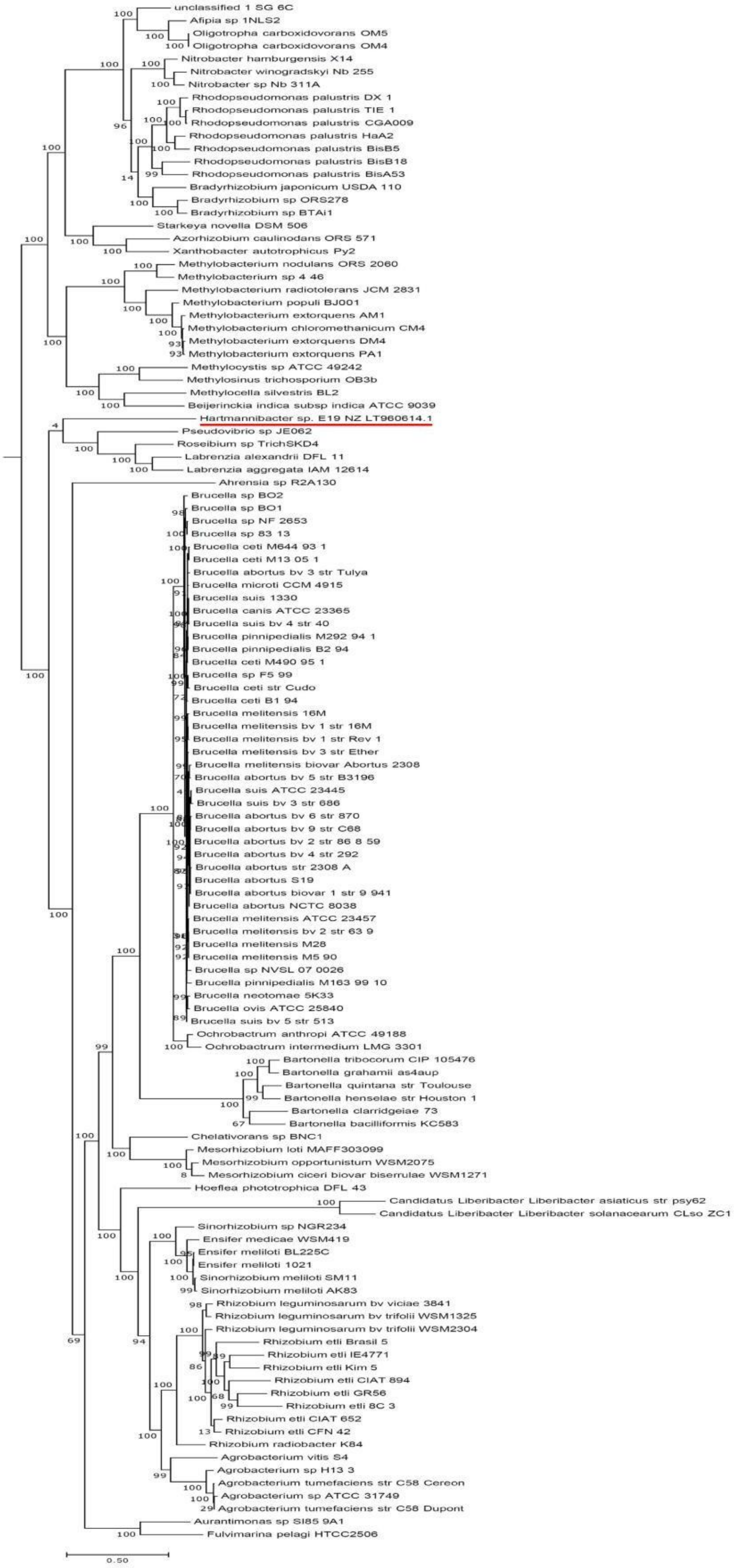

S2 Figure. Phylogenetic tree (neighbor joining algorithms) based on core genes sequences of strain E19^T and available closest gene sequence of members of related families of the orders *Rhizobiales* and *Rhodobacterales* and representatives of the class *Alphaproteobacteria*. Bootstrap values are shown in tree branches, when not shown values correspond to 100 %.

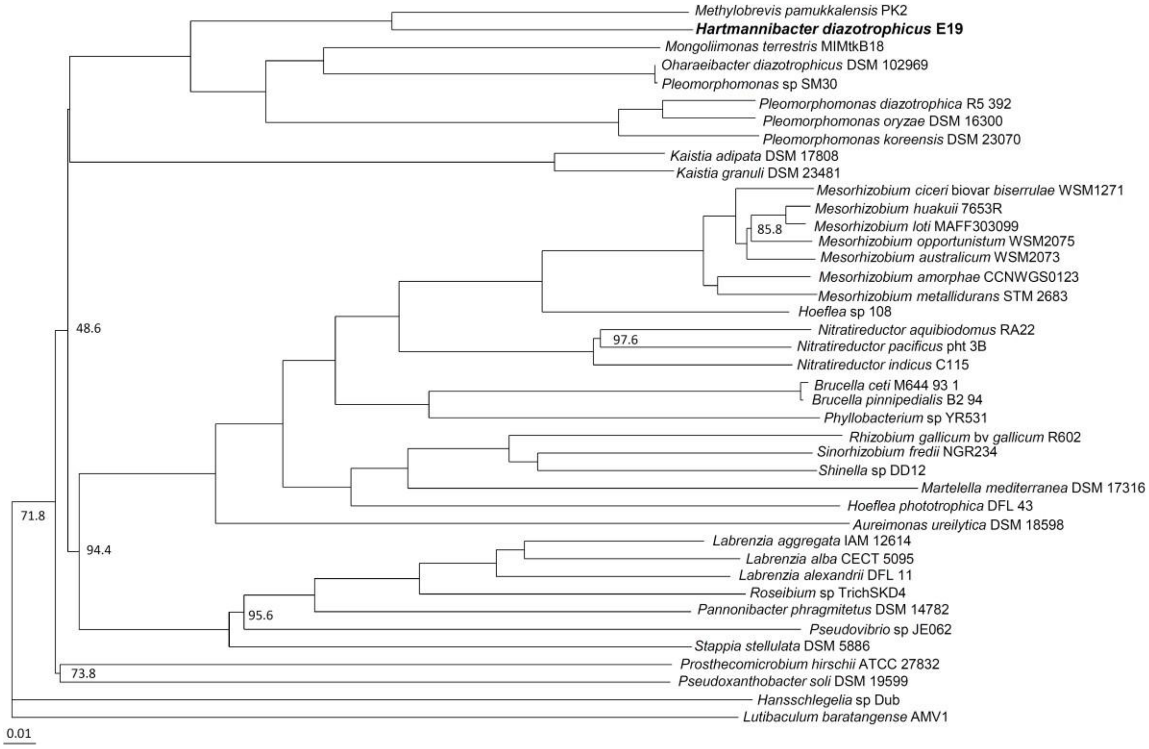

S3 Figure. Genomic organization of strain E19^{T} genome coding sequences for methanol dehydrogenase homolog genes and its closest relative genomes. Same arrow colors correspond at orthologous genes among genome sequences. A) CDS 2042, B) CDS 2631, C) CDS 3031.

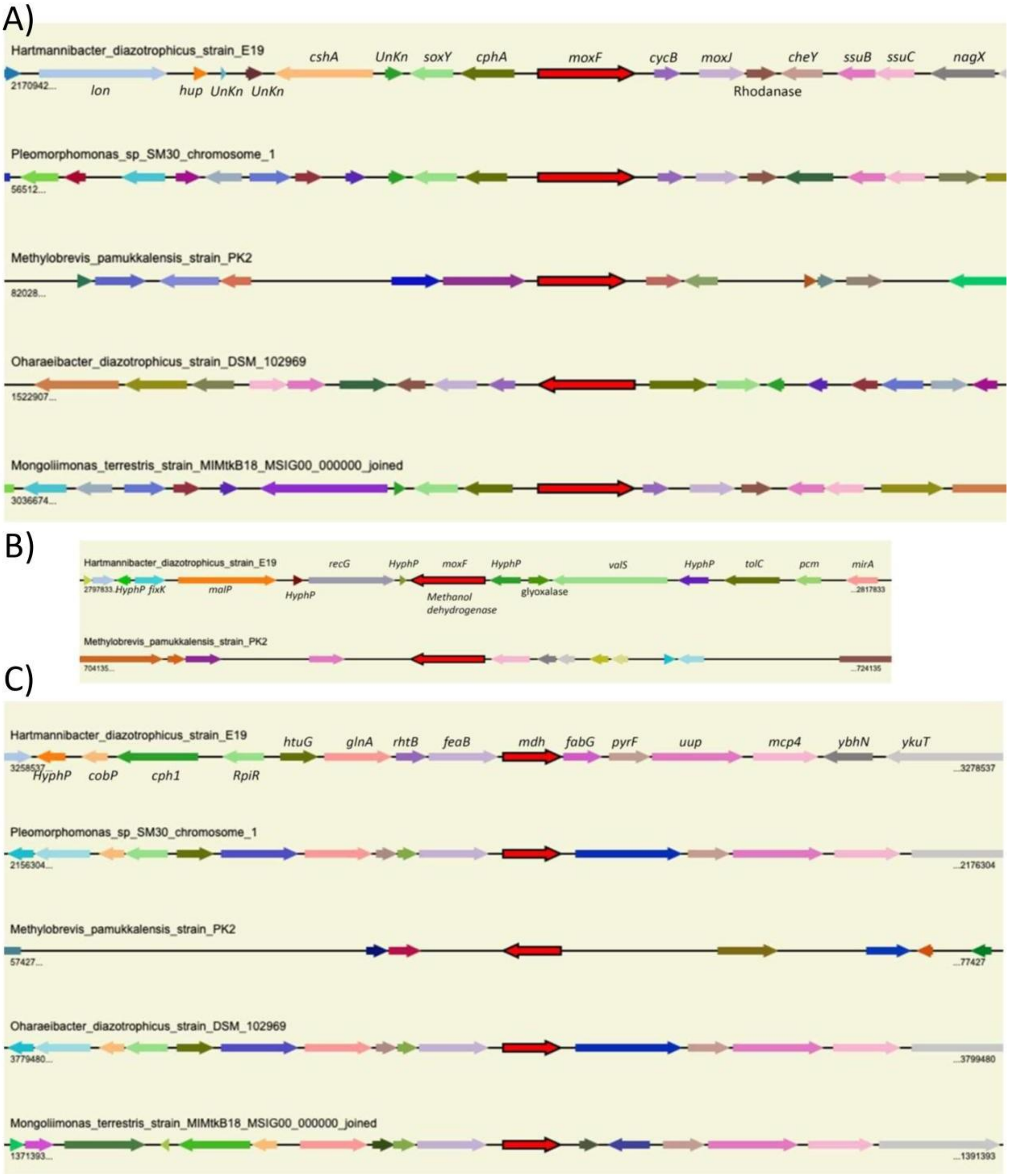

Supplement: Supplementary Materials — S1 Figure: precomputed phylogenetic tree of life integrating the H. diazotrophicus E19T genome calculated with PhyloPhlAn. Bootstrap values are shown in tree branches. S2 Figure: phylogenetic tree based on core gene sequences of strain E19T. Phylogenetic tree based on core gene sequences of strain E19T and the available closest gene sequence of members of related families of the orders Rhizobiales and Rhodobacterales and representatives of the class Alphaproteobacteria. Bootstrap values are shown in tree branches, when not shown values correspond to 100%. S3 Figure: genomic organization of the strain E19T genome coding sequences for methanol dehydrogenase homolog genes and its closest relative genomes. Same-color arrows correspond at orthologous genes among genome sequences. A) CDS 2042, B) CDS 2631, and CDS 3031. S1 Table: H. diazotrophicus E19T average nucleotide identity (ANI) and OrthoANI (average nucleotide identity by orthology) to the next relative available genome sequences. S2 Table: H. diazotrophicus E19T previously described phenotypical characteristics compared with its genomic content [11]. S3 Table: plant growth-promoting activities and respective genes present or absent in E19T. S4 Table: H. diazotrophicus E19T nitrogen fixation-related genes. S5 Table: H. diazotrophicus E19T phosphate-related genes. S6 Table: H. diazotrophicus E19T sulfur-related genes. S7 Table: H. diazotrophicus E19T CDSs containing PALP domain genes. S8 Table: H. diazotrophicus E19T methylotrophy-related genes. S9 Table: H. diazotrophicus E19T volatile organic compound-related genes. S10 Table: H. diazotrophicus E19T iron acquisition-related genes. S11 Table: H. diazotrophicus E19T salt tolerance-related genes. S12 Table: H. diazotrophicus E19T central metabolisms and protein secretory system-related genes. S13 Table: H. diazotrophicus E19T heavy metal resistance-related genes. S14 Table: H. diazotrophicus E19T aromatic compound degradation-related genes. S15 Table: H. diazotrophi [file 7586430.f1.zip › 7586430.Supp.pdf]
